# Molecular Biomarkers and Their Implications for the Early Diagnosis of Selected Neurodegenerative Diseases

**DOI:** 10.3390/ijms23094610

**Published:** 2022-04-21

**Authors:** Julia Doroszkiewicz, Magdalena Groblewska, Barbara Mroczko

**Affiliations:** 1 Department of Neurodegeneration Diagnostics, Medical University of Bialystok, 15-269 Bialystok, Poland; mroczko@umb.edu.pl; 2 Department of Biochemical Diagnostics, University Hospital in Białystok, 15-269 Bialystok, Poland; magdalena.groblewska@umb.edu.pl; 3 Department of Biochemical Diagnostics, Medical University of Bialystok, 15-269 Bialystok, Poland

**Keywords:** neurodegeneration, neuroinflammation, Alzheimer’s disease, Parkinson’s disease, amyotrophic lateral sclerosis, multiple sclerosis, molecular biomarkers, epigenetics

## Abstract

The degeneration and dysfunction of neurons are key features of neurodegenerative diseases (NDs). Currently, one of the main challenges facing researchers and clinicians is the ability to obtain reliable diagnostic tools that will allow for the diagnosis of NDs as early as possible and the detection of neuronal dysfunction, preferably in the presymptomatic stage. Additionally, better tools for assessing disease progression in this group of disorders are also being sought. The ideal biomarker must have high sensitivity and specificity, be easy to measure, give reproducible results, and reflect the disease progression. Molecular biomarkers include miRNAs and extracellular microvesicles known as exosomes. They may be measured in two extracellular fluids of the highest importance in NDs, i.e., cerebrospinal fluid (CSF) and blood. The aim of the current review is to summarize the pathophysiology of the four most frequent NDs—i.e., Alzheimer’s disease (AD), Parkinson’s disease (PD), amyotrophic lateral sclerosis (ALS), and multiple sclerosis (MS)—as well as current progress in the research into miRNAs as biomarkers in these major neurodegenerative diseases. In addition, we discuss the possibility of using miRNA-based therapies in the treatment of neurodegenerative diseases, and present the limitations of this type of therapy.

## 1. Introduction

Neurodegeneration is defined as the progressive degeneration of nerve cells, which results in their dysfunction and damage to their structure [1]. This process is irreversible, and may ultimately lead to cell death and the loss of neurons. Neurodegenerative diseases (NDs) are characterized by depositions of misfolded, toxic conformations of various proteins, which typically tend to aggregate into insoluble deposits. Moreover, NDs are multifactorial, and may also be associated with neuroinflammation and oxidative stress [2,3].

**Figure 1:**
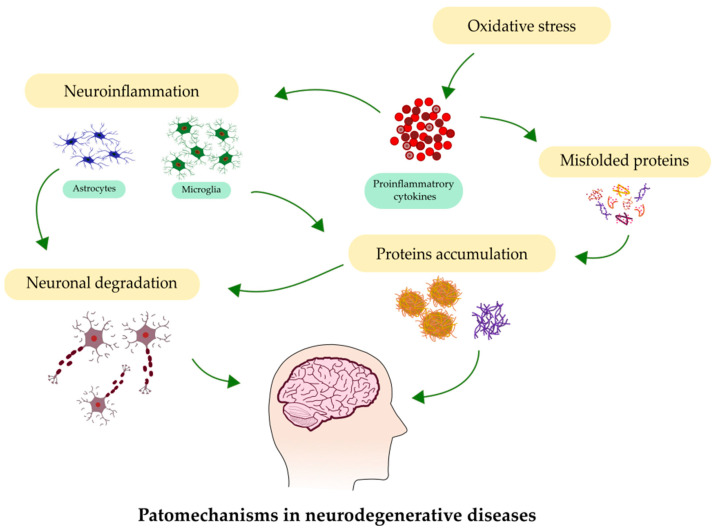
Most common processes involved in the pathology of neurodegenerative diseases.

Neurodegeneration, observed within the brain at various levels—from molecular to systemic—underlies many neurological disorders, such as Alzheimer’s disease (AD), Parkinson’s disease (PD), amyotrophic lateral sclerosis (ALS), and multiple sclerosis (MS). The number of people suffering from these disorders is increasing, mainly due to the aging of the population. The main processes involved in neurodegeneration are depicted in Figure 1. The early detection of NDs, at their initial stages, presents the highest chance to begin medical intervention. Moreover, the recognition of ND as early as possible at the onset of neurodegeneration can help to slow or even stop the progression of the disease.

Currently, the diagnosis of the majority of NDs is based on clinical examination supported by imaging techniques [4]. The main difficulty is that the evident clinical symptoms of NDs are most often found only after significant brain damage, the regeneration has occurred, and the ability to regenerate this damage is quite limited. Unfortunately, clinicians face difficulties in diagnosing these diseases before irreversible brain damage occurs, which is mainly due to the lack of effective diagnostic tools [5].

Thus, a search for noninvasive methods of disease detection is necessary. Widespread efforts are ongoing to identify biochemical markers circulating in biological fluids, allowing for quick, inexpensive, and specific identification of NDs, as well as their screening and staging [6]. The molecular diagnosis of neurodegenerative diseases is still in development, and must be further explored. It is believed that advancements in the earlier detection of these diseases will be helpful in their potential treatment, and could possibly even result in finding a cure.

Additionally, the use of miRNAs for therapeutic purposes in the treatment of AD and other neurodegenerative diseases has been investigated because of their involvement in multiple brain signaling pathways in various NDs. Therefore, we attempted to present miRNA-based therapeutic applications for the treatment of selected NDs. In addition, we discuss the limitations associated with the therapy of NDs, such as the permeability of the BBB, the bioavailability of miRNAs, and the various routes of administration of these drugs.

## 2. Molecular Biomarkers

As stated above, the term “biomarker” refers to a wide spectrum of molecules, from nucleic acids to proteins, peptides, lipids, metabolites, and other small molecules, and from small to large molecules, which can be detected with the use of genomics, proteomics, and other “-omics” technologies [7]. Bodily fluids comprise a variety of chemical molecular species, including nucleic-acid-based molecular biomarkers, such as gene mutations, polymorphisms, and quantitative gene expression analysis [8].

### 2.1. MicroRNAs

Circulating microRNAs (miRNAs) are an example of molecular biomarkers that may be useful in the diagnosis of neurodegenerative disorders. They are small strands of RNA, built with 21–22 nucleotides, which represent about 1% of human genes and do not encode proteins [9]. miRNAs are responsible for regulating post-transcriptional gene expression, which includes the growth of the cell, its development, and its programmed cell death, i.e., apoptosis [10]. These molecules are considered to be perfect biomarkers because of their ease of detection in many bodily fluids, including CSF and blood, with very high specificity in every biological material [11]. Moreover, miRNAs are characterized by high stability and the possibility of preservation—not only in formalin or paraffin, but also in frozen tissue material, which is not possible with larger RNA particles [12]. The release of miRNAs may occur both through passive discharge from damaged or dead cells (after apoptosis) and through active transport [13,14].

Many studies have linked miRNAs with the nervous system, and have discussed the possibility of regulating its development and functions using miRNAs [15]. miRNAs are involveded in synaptic plasticity and neurogenesis, and have a known influence on neuronal differentiation [14]. This has been confirmed in animal model research, in which the disturbed binding of miRNAs resulted in defects in healthy brain development [11,16]. Furthermore, after the injection of a certain group of miRNAs, there was a visible improvement in the healthy growth of animal models [16].

The majority of known miRNAs are expressed in the brain, but only some of them have been found to represent a brain-specific pattern of expression [17]. Research on the role of miRNAs within the central nervous system (CNS) has been conducted in order to ascertain their potential usefulness in various brain diseases, including NDs. The first analysis, which used next-generation deep sequencing (NGS) to contrast the profiles of miRNAs in cell-free CSF and serum in NDs, was also the largest dataset considering this topic [18]. Burgos et al. detected several miRNAs in blood and CSF obtained from patients with NDs, which had already been described in the brain samples obtained from patients suffering from these diseases [18]. Consequently, the dysregulation of miRNAs has been examined in neurodegenerative disorders that result in neuronal death, such as AD, PD, ALS, and MS [19,20,21].

### 2.2. Exosomes

Microvesicles—the smallest secreted organelles—that can be released from various cell types, including neurons, astrocytes, and microglia, in both healthy and diseased states, are called exosomes. They have been detected in and isolated from almost every known bodily fluid [22]. Exosomes originate from endosomes, and were initially described as useless waste from cells. However, it was recently stated that exosomes act as nanocapsules that have the ability to distribute a wide variety of molecules—such as proteins, lipids, and miRNAs—to direct targets [23].

It has been demonstrated that exosomes within the brain are able to mediate cell communication, but they can also be associated with the preservation of normal physiological processes in the brain, e.g., myelination [24]. Moreover, exosomes may be involved in some pathological conditions within the CNS, such as the transportation of APP metabolites in AD. Levels of amyloid β (Aβ) and tau protein were demonstrated to be elevated in exosomes obtained from AD patients’ brain samples [25]. Furthermore, similar results were obtained from experimental models of PD, with visible aggregation of α-synuclein transported by exosomes [26]. Although the exact role of exosomes is still not fully understood, these findings show the possibility of using them as potential biomarkers of neurodegenerative disorders, and should be further examined.

## 3. Alzheimer’s Disease

AD is the most common cause of dementia and the most widespread neurodegenerative disease [27]. It is a progressive disorder, with the first visible symptoms appearing as many as 20 years after onset [28]. It manifests with memory loss, the inability to learn new things, aphasia, disturbed sleep cycle, and significant problems with short- and long-term memory. In addition, AD patients require constant full-time care, which is an enormous burden not only for the families of the patients, but also for the healthcare systems [29]. Although research regarding AD is developing, and has been ongoing for many years, the exact pathology underlying the disease is still not fully known.

There are certain hallmarks of AD related to the pathology of this devastating disease. These include amyloid plaques—built with Aβ deposits—and tau protein, which accumulates in neurofibrillary tangles [30]. Another pathological hallmark of AD is neuroinflammation, which is linked to oxidative stress, triggered by various molecular compounds released as a result of inflammatory processes in the brain [31]. However, the etiology of AD is still not fully known and understood.

### 3.1. Genetic Mutations in AD

Although only about 3% of AD cases are familial, the genetics underlying the disease should be taken into consideration. The first discovered gene causing AD was *APP*, located on the 21q21 chromosome, and mutations of this gene are linked to early-onset Alzheimer’s disease (EOAD). Mutations within this gene may disturb the APP cleavage, resulting in Aβ aggregation.

Two other mutations involve the *PSEN1* and *PSEN2* genes, which are located at chromosomes 14q24.3 and 1q31–q42, respectively [32]. Proteins encoded by these chromosomes compose gamma-secretasean, which also participates in APP cleavage in an amyloidogenic pathway, leading to increased levels of Aβ42 [33]. Furthermore, *PSEN1* mutation is responsible for 80% of the cases of EOAD, while *PSEN2* mutation is responsible for only about 5% [32,34]. EOAD is common in patients with Down syndrome (DS), because in these patients with full trisomy 21, the *APP* gene is overexpressed, which makes DS the leading risk factor of the disease [35].

Another type of AD is late-onset Alzheimer’s disease (LOAD), which is related to the presence of alleles of the apolipoprotein E gene (*APOE*), localized on the 19q13.2 chromosome [34]. Four alleles of *APOE* have been identified. Furthermore, *APOE* has three leading isoforms: *APOE* ε2, ε3, and ε4. Among them, APOE ε4 is considered the major genetic risk factor for LOAD [32,36].

Interestingly, a polymorphism in APOE ε4 has been discovered in recent years, described as Klotho-VS heterozygosity, which in AD can lower the risk linked to APOEε4 carriers [36]. Klotho-VS heterozygosity in patients above 60 years of age was associated with a reduced risk of developing AD and progression from mild cognitive impairment (MCI)—defined as a stage between regular aging and AD [37]—to fully symptomatic AD. Furthermore more, these Klotho-VS heterozygotic patients had higher levels of CSF Aβ and a smaller amount of Aβ plaques in PET imagining, confirming the protective role of this polymorphism [38].

### 3.2. miRNAs and Other Molecular Biomarkers of AD

Currently, there are certain cerebrospinal fluid biomarkers that are considered to be diagnostic criteria for AD. These biomarkers are Aβ, total tau protein (tTau), and phosphorylated tau (pTau) [39]. It has been confirmed that Aβ may be released into biological fluids, such as CSF, during regular metabolism of APP, even when there is no known disability [40]. Furthermore, analysis of the amyloid plaques showed that they are composed mostly of Aβ1-40 and 1–42 isoforms. This implies that levels of circulating Aβ are likely to be decreased in AD patients in comparison to healthy controls, as has been confirmed by many researchers [41]. Moreover, it has been demonstrated that combined analysis of Aβ42 and Aβ40 in the CSF in the form of an Aβ42/Aβ40 ratio has a greater ability to detect AD than one biomarker on its own [42]. Another protein involved in AD pathology is tau. The main role of this protein is to maintain the stability of the microtubules. The disturbed function of tau results in synaptic dysfunction. Moreover, it has been revealed that phosphorylated tau protein is the major component of neurofibrillary tangles (NFTs) [39]. It was observed that CSF levels of tTau were elevated in AD patients, reflecting the severity of the disease. This confirms the diagnostic value of tTau as a biomarker of AD [43]. It has also been revealed that the phosphorylated form of tau is specific to AD—especially tau phosphorylated at threonine 181 (pTau181). It has also been shown that the simultaneous evaluation of Aβ and tau proteins may enhance their diagnostic specificity and sensitivity, making them the established panel of biomarkers for AD [39]. The measurement of these proteins in blood samples of AD patients was also considered, although they are not as accurate as CSF biomarkers, and must be compared to those already established [44].

A growing body of evidence demonstrates the deregulation of miRNA expression in AD patients. According to a meta-analysis by Swarbrick et al., several miRNA targets have been recognized, such as Aβ and tau signaling, inflammation, and apoptosis. However, most miRNA targets remain unknown [45]. It was shown that various miRNAs, such as miR-9, miR-29a, miR-29b, miR-34a, miR-125b, and miR-146a, may be expressed in mammalian brains [46]. Among these, the ones most regularly described are miR-9, miR-181, and miR-29 [47]. It was also revealed that some miRNAs, such as miR-29a and miR-29b-1—which are involved in the regulation of APP and beta-site APP-cleaving enzyme 1 (BACE1) expression—were decreased in AD brains. It has also been suggested that the loss of these specific miRNAs may contribute to the elevation of BACE1 and Aβ levels, leading to the abnormal formation of amyloid plaques in sporadic AD [48,49]. However, unlike brain studies, CSF analysis showed that miR-29a and miR-29b expression was increased, which might confirm that these molecules are released into the CSF from the tissues from which they originated [49]. Moreover, it has been proposed that the miRNAs described above may be considered as potential markers of AD [49].

The elimination of Aβ plaques—especially their degradation by various enzymes—may be helpful in reducing the onset of the disease. The degradation of these deposits may be connected to matrix metalloproteinase (MMP) activity—specifically MMP-2, which is expressed in activated astrocytes around plaques [50]. Cathepsin D is also involved in the degradation of amyloid plaques [51,52]. This enzyme is encoded by the *CTSD* gene, whose mutations may be involved in the pathogenesis of AD [53]. Moreover, in vitro upregulation of miR-128 resulted in the reduction in cathepsin D levels in cells obtained from AD patients. Furthermore, miR-128 blockage in monocytes from AD patients could also improve the degradation of Aβ_1–42_ [54]. Serum analysis showed significant upregulation of this miRNA in AD patients in comparison to controls. In addition, it was positively correlated with TNF-α and IL-1β levels [55].

The biological functions of miRNAs may be also connected to the regulation of tau protein—especially its phosphorylation. Chronic cerebral hypofusion (CCH) is one of the etiological factors for AD, which promotes the phosphorylation of tau proteins and contributes to AD progression. It has been demonstrated that CCH may also induce the deficiency of miR-132, which prevents the apoptosis of neurons, and is involved in the regulation of synaptic plasticity, learning, and memory [56,57]. The expression of this molecule was decreased in AD neurons characterized by the hyperphosphorylation of tau [58]. An experimental treatment with nimodipine resulted in the upregulated expression of miR-132 and attenuated CCH-induced tau hyperphosphorylation in a rodent model of AD [56]. This drug is a calcium channel blocker that inhibits the influx of Ca^2+^ into smooth muscle cells and prevents calcium-dependent vasoconstriction. Another effect of nimodipine treatment is the inhibition of GSK-3, which is one of the main protein kinases involved in the phosphorylation of the tau protein [56]. Therefore, miR-132 is considered to be neuroprotective, and it has shown the ability to improve cognitive functions in rodent models of AD [59,60]. Another miRNA molecule, miR-124-3p, has been described as being able to lower the hyperphosphorylation of the tau protein in cell cultures, via the PI3K/AKT/GSK-3 pathway [61].

Although miRNAs regulate several genes involved in oxidative stress response and neuroinflammation, oxidative stress itself can also alter the expression of miRNAs. Moreover, Aβ may induce the formation of reactive oxygen species (ROS), leading to oxidative stress [62]. It has been revealed that miR-125b may promote the expression of APP and BACE1, as well as the production of Aβ, in an in vitro AD model [63]. In addition, miR-125b induced apoptosis and inhibited the proliferation of cells, as well as enhancing oxidative stress and inflammation [63]. In other research, synthetic soluble Aβ promoted miR-134, miR-145, and miR-210 expression in neuronal models [64].

Importantly, disturbed miRNAs may be observed not only in fully developed AD, but also at earlier stages, in patients with MCI diagnosis. Sheinerman et al. described two sets of miRNAs in the plasma of MCI patients, namely, the miR-132 family—consisting of miR-128/miR-491-5p, miR-132/miR-491-5p, and mir-874/miR-491-5p—and the miR-134 family, consisting of miR-134/miR-370, miR-323-3p/miR-370, and miR-382/miR-370, which is characterised by very high specificity and sensitivity [65]. In addition, the identified pairs of miRNA biomarkers could already detect MCI in patients at the asymptomatic stage, 1–5 years prior to clinical diagnosis [14,65]. Another study by Nagaraj et al. showed that hsa-miR-483-5p and hsa-miR-486-5p were elevated, and had the highest statistical significance of the miRNAs that they assessed in blood, in terms of biomarkers of MCI, and even later stages of AD [66].

Furthermore, significant changes in miRNAs—specifically miR-29c, miR-136-3p, miR-16-2, miR-331-5p, miR-132-5p, and miR-485-5p—were found in exosomes of CSF obtained from AD patients in comparison to healthy controls [67]. McKeever et al. demonstrated a decline in exosomal miR-16-5p, miR-451a, and miR-605-5p, and an increase in miR-125b-5p, in the CSF of EOAD patients compared with healthy controls [68]. In addition, they analyzed material obtained from a group of LOAD patients, and showed reduced miR-451a and miR-605-5p, with no differences in miR-16-5p, compared to healthy controls [68]. These results suggest that miR-16-5p can be considered as a potential CSF biomarker of EOAD [20].

The diagnostic utility of exosomes as blood-based biomarkers has also been analyzed. Several works have described potential panels of exosomal miRNAs dysregulated in AD. For instance, Lugli et al. defined seven miRNAs—miR-342-3p, miR-141-3p, miR-342-5p, miR-23b-3p, miR-24-3p, miR-125b-5p, and miR-152-3p—as significant predictors of AD in a machine learning model of AD, with miR-342-3p having the most statistically significant results [69]. In addition, Yang et al. analyzed miR-135a, miR-193b, and miR-384 in patients with MCI, AD–dementia, PD with dementia, and vascular dementia, in order to demonstrate the potential utility of these miRNAs in AD diagnosis [70]. They revealed the upregulation of miR-135a and miR-384 but downregulation of miR-193b in the sera of AD patients. Importantly, miR-384 was the best in terms of the differentiation between the examined groups [70]. Table 1 summarizes the chnges characteristic of AD.

## 4. Parkinson’s Disease

PD is the second most prevalent neurodegenerative disease in the world [72]. It is a progressive motor disease in which the nigrostriatal dopaminergic pathway is degenerated. However, both motor and nonmotor neurons are affected in this disease. Motor symptoms include bradykinesia, resting tremor, stiffness of the limbs, and postural instability, with problems with balance and coordination [73]. Nonmotor symptoms, which can even foreshadow the known movement disturbances, consist of sleep disturbances, hyposmia, depression, and constipation [74].

One of the histopathological hallmarks of PD is an aggregation of misfolded, insoluble α-synuclein, which has the ability to form Lewy bodies, and the development of abnormal Lewy neurites in diseased neurons, which contain abnormal α-synuclein filaments similar to those found in Lewy bodies, which promote neurodegeneration [75]. Similar to AD, PD may also be linked to neuroinflammation, with known micro- and astrogliosis [76].

### 4.1. Genetic Mutations in PD

In terms of the aetiology of PD, approximately 5–10% of the cases may be caused by genetic factors, with autosomal dominant or recessive inheritance. Some monogenic forms of PD mutations have been identified. In the late-onset form of the disease, these are the α-synuclein (*SNCA*) and leucine-rich repeat kinase2 (*LRRK2*) genes, while in early-onset PD, they are are parkin (*PARK2*), PTEN-induced putative kinase 1 (*PINK1*), and oncogene DJ1 (*DJ1*) [14].

In order to develop PD, autosomal dominant forms need mutations in two genes. Mutations in the *SNCA* gene are uncommon, fully penetrant, and include point mutations and duplications or triplications, with copious amounts of Lewy bodies in the brain [77]. On the other hand, *LRRK2* mutations are the best known source of autosomal dominant PD, with variable penetrance [78]. They cover around 10% of patients with familial PD, and the clinical manifestation of the disease is very similar to idiopathic late-onset PD [77,79].

On the other hand, PD cases with autosomal recessive mutations are more frequent. Mutations in the parkin gene are the most common, and comprise 50% of disease manifestation before 45 years of age, and around 10–20% of early-onset PD [77,80]. *PINK1* and *DJ1* mutations have similar manifestations as parkin mutations, with slow progression, early motor symptoms, and nonexistent nonmotor symptoms [81]. Furthermore, the *PINK1* mutations are strictly connected to the earliest types of PD [77].

### 4.2. miRNAs and Other Molecular Biomarkers of PD

Although there is no accepted diagnosis for PD based on biochemical analysis of blood or CSF, α-synuclein is one of the best-studied biomarkers of this disease. This protein plays a significant role in the pathology of PD, with a known ability to aggregate into Lewy bodies. Meta-analyses of α-synuclein in CSF showed decreased levels of this protein in PD patients compared to healthy controls, but also compared to other neurological disorders [82,83]. Not only has total α-synuclein been studied, but so have other types of this protein, such as its oligomeric and phosphorylated forms. Unlike total α-synuclein, the oligomeric form of this protein was found to be elevated in the CSF of PD patients in comparison to healthy controls, although it did not have high diagnostic sensitivity [82]. In addition, the simultaneous assessment of total and oligomeric synuclein, along with a calculated ratio of the oligomeric form to total protein, improved their diagnostic usefulness, but it was still not tolerable in clinical practice [84]. Similar results were obtained when considering phosphorylated synuclein [85]. CSF levels of this form of α-synuclein were also elevated in PD compared to HC. Unfortunately, the measurement of phosphorylated α-synuclein in the blood has several limitations, because erythrocytes are the major source of this protein in the blood [86]. The DJ1 protein is another potential biomarker of PD that can be evaluated in CSF and blood. Elevated levels of this protein were observed in PD patients, which may reflect ongoing oxidative stress processes [87]. However, the measurement of this protein in blood samples may be affected by its high load in erythrocytes [88].

The determination of fluid biomarkers in PD has restricted their usefulness, because of many methodological issues related to their detection and quantification. Therefore, researchers’ attention has shifted to molecular biomarkers. Briggs et al. found upregulated expression of miR-132, miR-92a, miR-27a, and miR-148a, while miR-744 and miR-532-5p were found to be downregulated, in brain samples of PD patients [89].

Moreover, there is a link between the accumulation of α-synuclein in PD brains and changes in miRNAs’ expression [90]. It has been shown that α-synuclein can be altered by at least four miRNAs: miR-7, miR-153, miR34b/c, and miR-214. Oxidative stress—a major mechanism in the pathogenesis of PD—might influence α-synuclein levels via miR-7 or miR-153 inhibition, influencing the accumulation of this protein in the brain [91].

Furthermore, the analysis of circulating miRNAs that are present in patients’ blood also reveals their altered expression. Margis et al. compared blood samples obtained from treated and untreated PD patients, and demonstrated the downregulation of miR-1, miR-22*, and miR-29a, as well as the upregulation of miR-16-2*, miR-26a-2*, and miR-30a, in PD patients without any treatment [92]. Interestingly, miR-1, miR-22-5p, and miR-29a levels also differed significantly in PD patients in comparison to healthy controls [92]. Moreover, the analysis of miRNA profiles showed the upregulation of miR-331-5p [66,93]. Another study described a panel of PD-predictive biomarkers, including miR-1826, miR-450b-3p, miR-626, and miR-505, in the blood of PD patients, with 100% specificity and 91% sensitivity [94].

As with AD, exosomal miRNAs were studied in CSF obtained from PD patients. Gui et al. showed that miR-1 and miR-19b-3p in exosomes were significantly downregulated in these patients, while exosomal miR-153, miR-409-3p, miR-10a-5p, and let-7g-3p were upregulated [67]. The changes and characteristics described above for PD are summarized in Table 2.

## 5. Amyotrophic Lateral Sclerosis

ALS is another neurodegenerative disease affecting motor neurons, with a fatal prognosis. The approximate survival after the onset of symptoms is 2–5 years in the majority of ALS patients, with faster progression of the disease in the elderly. However, 5% of patients survive up to 20 years [98]. ALS is characterized by the progressive loss of both upper and lower motor neurons, which causes muscle stiffness, spasticity, and twitching. As the disease progresses, the neuromuscular connections are gradually lost, resulting in muscular atrophy at the stage of full degeneration [96]. Moreover, half of patients present some degree of cognitive impairment [97].

Similarly to other NDs, ALS is also characterized by the accumulation of misfolded proteins, such as transactive response DNA-binding protein 43 kDa (TDP-43), which is modified in several pathological processes, such as phosphorylation and mislocalization, and this also occurs in the motor neurons [99]. Other frequently aggregating proteins in ALS are wild-type superoxide dismutase 1 (SOD1) and ubiquilin 2 [100,101]. In addition, neurodegeneration is accompanied by neuroinflammation, occurring with microgliosis and astrogliosis [96,102].

### 5.1. Genetics of ALS

In approximately 10% of all ALS cases, the disease can be attributed to genetic background, mostly with dominant gene mutations; this is known as familial ALS. The first described gene whose mutations are associated with ALS was *SOD1*, located on chromosome 21 [97], which encodes SOD1—an enzyme responsible for protecting the cells from reactive oxygen species through the catalyzation of the dismutation of superoxide anions to oxygen and hydrogen peroxide [103]. It has been shown that ALS-causing *SOD1* mutations act mainly in a dominant fashion, and a single copy of the mutant *SOD1* gene is sufficient to cause the disease [104]. However, the exact molecular mechanisms by which *SOD1* mutations cause disease are not fully understood.

A more common genetic abnormality related to ALS is the mutation of C9orf72 hexanucleotide repeat expansion, which may contribute to approximately 40% of familial ALS cases. This mutation causes increased toxicity of C9orf72—a protein found in brain, motor, and nonmotor neurons, resulting in the loss of their function [105].

*TARDBP* is another gene involved in the pathology of ALS. This gene encodes TDP-43—a protein responsible for RNA metabolism in normal conditions [103]. It has been demonstrated in mice that TDP-43 also plays an important role in embryonic development, as well as in later life, influencing the control of muscle movement [106,107]. In ALS, TDP-43 is most commonly found in its hyperphosphorylated form, with a high tendency to aggregate in the cytoplasm of motor neurons [97,103]. In addition, nuclear factor κB (NF-κB) and type I interferon pathways participate in processes of neurodegeneration mediated by TDP-43, and increase the production and release of other proinflammatory cytokines [108]. Furthermore, more than 50 mutations were recently described, which manifest mostly as cognitive impairment in ALS patients [109].

### 5.2. miRNAs and Other Molecular Biomarkers of ALS

The diagnosis of ALS is mostly based on clinical examination, existing symptoms, and available tests to exclude other diseases [12]. Due to the large number of genes involved in the development of ALS, it is so far not possible to find a biomarker with 100% specificity and diagnostic sensitivity for the diagnosis of this disease. However, certain ALS biomarkers have been developed. CSF biomarkers of ALS can be divided into those reflecting ongoing neuroinflammatory processes and those reflecting neuronal loss [14].

Neurofilaments, which are mainly found in neurons, were previously linked to the pathology of ALS [110]. They are released primarily into the CSF, and later into the blood, in response to axonal injury. Initial studies described higher concentrations of phosphorylated neurofilament heavy chains (pNfH) in ALS patients in comparison with healthy controls [110,111]. Newer research has described high diagnostic specificity and sensitivity of pNfH in ALS patients compared with controls, suggesting that pNfH could be a potential biomarker of the disease [112,113].

Neurofilament light chains (Nf-L) are another type of neurofilament, and are currently considered the most advanced biomarker for ALS [114]. The concentrations in both CSF and blood were higher in ALS patients in comparison to healthy controls and other patients without CNS involvement [114]. However, due to their low disease specificity, measurements of CSF Nf-L should instead be considered as a biomarker of disease severity, while blood Nf-L should be used as a biomarker of the progression of the disease—not strictly for the confirmation of ALS [115,116].

As described previously, neuroinflammation plays an important role in the pathology of ALS. Therefore, proteins involved in inflammatory response could also be investigated as potential biomarkers of ALS. A meta-analysis conducted by Hu et al. revealed that blood levels of inflammatory proteins such as TNF-α, IL-6, IL-1β, IL-8, and VEGF were significantly higher in ALS patients when compared with healthy controls [117].

Although there are few studies regarding the role of miRNAs in ALS, Freischmidt et al. analyzed sera from familial and sporadic cases of ALS. They discovered that different miRNAs are deregulated in different types of the disease. Patients with familial ALS showed downregulation in four miRNAs—miR1915-3p, miR3665, miR4530, and miR4745-5p—whereas in patients with the sporadic type of the disease these molecules presented regular expression, with the exception of downregulated miR1234-3p and miR1825 [118,119]. Importantly, the authors showed that in asymptomatic ALS-related mutation carriers, there was visible downregulation of miRNAs, which forecasted the full manifestation of the disease in the following 20 years [118]. This evidence may suggest the presence of these biomarkers even before the full manifestation of the disease, allowing for its early diagnosis.

On the other hand, Waller et al. showed the decreased expression of miR374B-5p and increased expression of miR143-3p and miR206 in sera; the latter two were increasingly dysregulated over time, suggesting a connection between these miRNAs and the progression of the disease [120]. However, researchers suggest that using a single molecule for identifying and monitoring the disease is not efficient, and panels of such molecules should be established to obtain the best results. However, there are not many studies regarding this issue, and the theme must be deeply analyzed. A summary of changes characteristic of ALS is shown in Table 3.

## 6. Multiple Sclerosis

Multiple sclerosis is a chronic demyelinating, inflammatory, and neurodegenerative disease, which may be caused by an autoimmune response [121]. It particularly affects adults between 20 and 40 years of age, with a visible predominance in women, which is not typical for the previously described diseases [122]. The pathology of MS is still not fully understood; however, it is postulated that activated T and B cells cross the blood–brain barrier (BBB), entering the CNS, where they exert inflammatory effects against the CNS—especially the myelin sheath [123]. There is a strong connection between environmental factors and the development of the disease. The most known are Epstein–Barr virus (EBV) infection and vitamin D deficiency [124]. Patients who were infected with EBV in childhood and in adolescence have a 15- and 30-fold higher chance of developing MS, respectively [125].

There are four main types of MS: clinically isolated syndrome (CIS), relapsing–remitting MS (RRMS), secondary progressive MS (SPMS), and primary progressive MS (PPMS). Among them, the most common is RRMS [126]. Although the course of MS can vary between patients, some characteristic patterns of the disease may be observed. First, MS attacks are mild or even asymptomatic, and are only later considered as incidents of MS. Further development of the disease may appear as a gradual worsening of the symptoms, or in the form of relapses followed by periods of improvement [127]. Most patients have typical neurological symptoms, such as double vision, muscle weakness, and numbness in the limbs, as well as chronic pain and speech problems [128].

### 6.1. Genetic Mutations Involved in MS

Although MS is not an inherited disease, there is a certain known link to genes in the pathology of this disease. Genetic cases cover around 30% of MS patients [129]. The first mutation discovered in patients was connected to the human leukocyte antigen (HLA) [130]. The closest allele related to MS susceptibility in Caucasians is HLA-DRB1*15:01 [131,132]. Nevertheless, the absence of this haplotype does not guarantee avoiding the disease, because 40% of Caucasians are not its carriers. Furthermore, in other ethnicities, there are different haplotypes linked to MS [132]. Another gene, discovered more recently, is *IL7RA*, located on chromosome 5p13.2, and its polymorphisms. This gene encodes IL-7RA, which is expressed by lymphocytes, but can also be found in dendritic cells [133,134].

### 6.2. miRNAs and Other Molecular Biomarkers of MS

Confirmation of the disease is achieved with oligoclonal bands and immunoglobulins (IgGs), which are analyzed simultaneously in the blood and CSF via the isoelectric focusing method [135]. In MS, IgGs are produced intrathecally by plasmatic cells, although other immunoglobulin classes—i.e., IgA and IgM—may also be synthesized [136]. The disease is confirmed with the presence of at least two oligoclonal IgG bands in the CSF but not in the serum, as has been described in almost every patient with established MS [137]. Concentrations of IgG in the CSF should also be analyzed concurrently with the measurement of these immunoglobulins’ levels in the patient’s serum. These results should be presented as an IgG quotient (Q_IgG_) [126], which describes the intrathecal production of immunoglobulin G [127].

In addition to immunoglobulins, plasmatic cells infiltrating the BBB are also capable of producing immunoglobulin-free light chains (FLCs) of either kappa (KFLC) or lambda (LFLC) chains, which may be analyzed in both the CSF and the serum [138,139]. Levels of KLFC in MS patients are increased, and are characterised by high diagnostic sensitivity [140].

Similarly to ALS, concentrations of neurofilaments were also examined as possible biomarkers of MS. Disanto et al. described elevated levels of NfL in the blood of MS patients compared to a healthy control group [141]. Moreover, in treated patients, levels of this molecule decreased over time, so NfL should be considered in the monitoring of patients during treatment [142].

Inflammatory cytokines were also investigated as potential biomarkers of MS. Huang et al. described elevated levels of the CSF-to-serum quotient of some inflammatory proteins, such as IL-12B, CD5, eotaxin-1, MIP-1a, and CXCL9, which showed results similar to IgG assessment [143]. Marking some of them (CCL11 and CCL20) showed a connection to disease progression and severity [143]. This revelation suggests the potential usefulness of inflammation biomarkers in monitoring the disease and predicting its severity.

miRNAs may also be analyzed in MS patients. It was demonstrated that miRNAs may be involved in immunity, inflammation, and neurodegeneration. Moreover, the dysregulation of certain miRNAs was also observed in MS, but only a few of them were directly related to disease activity [144]. Some miRNAs stimulate various immunological processes. For example, miR-125a may be involved in macrophage activity and diversification of B cells, while miR-146b is widely expressed in various immunocyte lineages—such as IL-17-producing T cells, Treg cells, monocytes, dendritic cells, macrophages, and B cells—participating in their activation and differentiation. Another miRNA, miR-200c, suppresses the expression of the Ets-1 protein—a negative regulator of Th17 differentiation [145].

Yang et al. discovered that, in Chinese patients, blood expression of miR-125a, miR-146b, and miR-200c was higher, while that of miR-328, miR-199a, and miR-152 was lower, in MS patients compared to healthy controls [145]. A more recent meta-analysis described 11 upregulated miRNAs in MS subjects: miR-145, miR-376 c-3p, miR-128-3p, miR-191-5p, miR-26a-5p, miR-320a, miR-486-5p, miR-320b, miR-25-3p, miR-24-3p, and miR-140-3p. On the other hand, eight of the molecules found were downregulated: miR-572, miR-15b, miR-331-5p, miR-23a, let-7 c-5p, miR-16, miR-24, miR-137, and miR-181 [146]. However, the levels of these miRNAs varied between different types of MS; miR-145, miR-223, miR-128-3p, and miR-191-5p showed high sensitivity and specificity in terms of the potential usefulness of these molecules [146]. Perdaens et al. analyzed miRNAs with known differentiation, according to the status of the disease (in relapse or remission) [147]. Regardless of the disease activity, MS patients had a higher expression of miR-150-5p and miR-155-5p, while miR-15a-3p and miR-34c-5p were lower, compared to controls. Moreover, among different groups of patients, miR-20a-5p, -33a-3p, and -214-3p were downregulated among those in remission, while miR-149-3p was upregulated, in comparison to relapsed patients and controls [147]. However, these are initial studies, and there is still a need for further analysis. A summary of the characteristic miRNA changes in MS is shown in Table 4.

## 7. An miRNA-Based Therapeutic Approach to NDs

In recent times, biopharmaceuticals based on miRNAs have emerged as one of the essential interests in next-generation medicine [148]. Systemically delivered miRNAs may overexpress the transcript by acting as miRNA mimics, or they can silence the function of the transcript as miRNA repressors. The mode of miRNA action is multi-targeted [149]. While a single miRNA is able to bind several transcripts, one mRNA can also be regulated by numerous miRNAs simultaneously, resulting in a relative endogenous abundance of a given molecule. Therefore, miRNA-related studies call for extensive preclinical assessment [150,151,152].

Moreover, changing the expression of specific miRNAs can influence the drug sensitivity and modulate the resistance to standard therapies for cancer [153]. These miRNA-based drugs can be administered intravenously or via injections. Additionally, the intratumoral route of administration may be used in the case of cancer-related pathologies, which allows for increasing target specificity and efficacy, and can minimize side effects [154,155]. There are ongoing clinicial trials using various miRNAs in the treatment of various diseases, including malignant tumors and leukemias [156]. Additionally, this approach has been tested in a variety of nonmalignant diseases, including myocardial infarction, arrhythmia, heart failure, acute lung injury, lung fibrosis, liver fibrosis, chronic hepatitis B and C, pancreatitis, diabetes and diabetic nephropathy, osteoporosis, and keloid formation [157].

Importantly, there have also been attempts to use this type of treatment in certain neurodegenerative diseases, such as AD and PD. The knowledge of outcomes regarding miRNAs suggests that altering their expression might be beneficial to the disease onset.

In several AD mice models, the direct delivery of miR-132 synthetic mimics into the brains of these animals restored the levels of native miR-132, which resulted in the improvement of memory deficits and normalization of tau metabolism [153,158,159]. Furthermore, it was demonstrated that injections of lentiviral constructs expressing miR-338-5p in the 5xFAD murine model of AD resulted in the overexpression of this miRNA and decreased BACE1 levels, Aβ formation, and neuroinflammation [160]. Similar results were obtained in APP/PS1 after intranasal miR-146a distribution [161]. These findings suggest that regulating miRNA expression may be considered a therapeutic approach to the diseases, although these are only in vitro studies, from which conclusions cannot be easily drawn.

In contrast to diseases located outside the central nervous system, one of the essential problems in the implementation of miRNAs as a medication for NDs is the route of administration of these putative drugs. In the case of experimental therapies, animals can be given drugs directly into the brain—specifically into areas affected by disease; in humans, however, such treatment is not possible. Therefore, these problems with the method of delivery of drugs to the brain have been investigated for decades, although with variable effectiveness. Apart from intravenous injections, one of the acceptable delivery methods in humans is intranasal delivery [162,163]. The administration of drugs into the CSF could be another solution. However, this is an invasive procedure, with significant risks.

Another issue in the design of drugs for NDs is that they must be able to cross the BBB and penetrate the brain after intravenous administration. In normal conditions, without ongoing inflammation, this is possible only for lipid-soluble small molecules—presumably smaller than 400 daltons—while macroparticles cannot successfully penetrate this barrier. Due to the limited ability to administer drugs into the brain because of the need to cross the BBB, new possible methods of distribution have been researched. The proposed solutions include nanoparticles, liposomes, and modified micelles.

Another approach assumes the use of effective virus-based vectors, such as viral recombinant adeno-associated virus (rAAV)-based systems expressing miRNAs [158]. These have already been used in murine AD models, although injected intracranially. It has been demonstrated that miR-124–3p administration resulted in a signficant reduction in Aβ deposits and promoted improvements in cognitive functions in these animals [159]. Moreover, in murine ALS models, intrathecal delivery of AAV-encoding miRNAs, targeting the *SOD1* gene, leads to its silencing, as well as to activated peripheral immune response [149,164].

Exosomes may constitute an alternative route of administration of potential miRNA-based drugs for NDs. They are capable of crossing the BBB, and may act as transportation instruments. In a study by Qu et al., exosomes filled with dopamine were able to cross the BBB in murine models of PD, showing better therapeutic effects and less toxicity than typical intravenous distribution [165]. Moreover, exosomes derived from mesenchymal stem cells (MSCs) were also described as potential carriers of the useful miRNAs in animal models of PD. miR-133b transmitted by MSC exosomes was delivered to neuronal cells, where it promoted neurite outgrowth. Furthermore, the inhibition of α-syn aggregation and suppressed NLRP3 activation was observed in animal models of PD when modifying MSC-derived exosomes with mimic-miR-7 [166]. Interestingly, exosome modification with antago-miR-155 can result in reduced microgliosis and ongoing neuroinflammation [166].

However, studies concerning the potential use of miRNA as a treatment for NDs are still in the preclinical stage. To the best of our knowledge, such formulations have not reached clinical trials, nor have any of the miRNA-based drugs been approved and introduced for the treatment of these diseases so far.

## 8. Conclusions and Future Perspectives

In the present review, we describe the current knowledge concerning the main genetic disturbances and the molecular biomarkers for the early detection and diagnosis of selected NDs. We discuss genes, molecular markers, and specific proteins that have been identified in recent years, in the areas of both genetic and biochemical molecular markers. Molecular biomarkers, such as circulating miRNAs, may provide new insights into the diagnosis and monitoring of specific neurodegenerative disorders, such as AD, PD, ALS, and MS. The evidence reviewed here suggests that the analysis of miRNAs reveals them to be a highly promising “perfect biomarker” for neurodegenerative disorders.

Moreover, the advances in the analyses of circulating miRNAs described in our paper might lead to a more efficient effort toward new biomarkers for NDs, in order to facilitate the identification of new therapeutic targets. In recent years, the knowledge regarding miRNAs has led to the conclusion that altering their expression might also be beneficial for the possible treatment of some NDs—especially at the onset of the disease. Therefore, in this paper, we discuss the possibility of using miRNA-based therapies in the treatment of neurodegenerative diseases, and present the limitations from preclinical studies associated with the use of this type of therapy prior to entering clinical practice.

In conclusion, the present paper reviews the literature to summarize the knowledge of microRNA regulation in the pathophysiology of selected neurodegenerative and neuroinflammatory diseases, and to discuss how these discoveries can be exploited for the development of microRNA-based therapies. It seems that miRNAs are promising tools both as novel biomarkers and in the treatment of these diseases. However, further studies are required to develop miRNAs for the clinical diagnosis and therapy of NDs.

## Figures and Tables

**Table 1:** AD biomarkerss.

Sample Type	miRNA Tested	Expression	Influence	Study Group	Author
Brain tissue	miR-29a, miR-29b-1	↓	Regulation of APP and beta-site APP-cleaving enzyme 1 (BACE1)	Adult murine and human organs	[46]
CSF	miR-29a, miR-29b	↑		10 AD 10 CTRL	[49]
Blood cells	miR-128	↑	Reduction in cathepsin D levels in monocytes	34 AD 37 CTRL	[54]
Blood	hsa-miR-483-5p and hsa-miR-486-5p	↑	Direct ERK1/2 repression lowering phosphorylation of tau	13 AD 8 MCI 9 CTRL	[66,71]
CSF exosomes	miR-29c, miR-136-3p, miR-16-2, miR-331-5p, miR-132-5p, miR-485-5p	↓	Regulation of beta-site APP-cleaving enzyme 1 (BACE1)	28 AD 27 CTRL	[67]
	miR-16-5p, miR-451a, miR-605-5p	↓		13 LOAD 17 YOAD 12 CTRL	[68]
	miR-125b-5p	↑			
Blood exosomes	miR-135a, miR-384	↑	Repression of BACE-1 and/or APP expression and activity	101 MCI 107 AD	[70]
	miR-193b	↓			

AD—Alzheimer’s disease patients; CTRL—normal controls; MCI—patients with mild cognitive impairment; LOAD—late-onset Alzheimer’s disease patients; YOAD—young-onset Alzheimer’s disease patients.

**Table 2:** Molecular biomarkers of PD.

Sample Type	miRNA Tested	Expression	Influence	Study Group	Author
Brain tissue samples	miR-132, miR-92a, miR-27a, miR-148a	↑	Enhancing the activation of microglial cells and the loss of microglia cells by mediating GLRX	8 PD 8 CTRL	[89,95]
	miR-744, miR-532-5p	↓	Upregulation of retinoic acid receptor alpha (RARA) and teratocarcinoma-derived growth factor 1 (TDGF1) genes		
	miR-132-3p	↑	Activation of microglial cells	5 PD 5 CTRL	[95]
Blood	miR-1 ^&^, miR-22*, miR-29a	↓	^&^ Regulating the fas-apoptotic inhibitory molecule (FAIM), death receptor antagonist	15 PD 8 CTRL	[92,96,97]
	miR-16-2*, miR-26a-2*, miR-30a ^&^	↑	^&^ Regulating the ubiquitin-mediated degradation of glutamate transporter 1 (GLT-1)		
	miR-1, miR-22-5p, miR-29 ^&^	↓	^&^ Neuronal survival, proliferation, differentiation, and plasticity		
	miR-331-5p	↑	NA	31 PD 25 CTRL	[66,93]
	miR-1, miR-19b-3p	↓	NA	47 PD 27 CTRL	[67]
	miR-153, miR-409-3p, miR-10a-5p, let-7g-3p	↑	NA		
CSF exosomes	miR-1, miR-19b-3p	↓	NA	47 PD 27 CTRL	[67]
	miR-153, miR-409-3p, miR-10a-5p, let-7g-3p	↑	NA		

PD—Parkinson’s disease patients; CTRL—normal controls; NA—not assessed; ^&^—The influence corresponds only with marked miRNAs.

**Table 3:** Molecular biomarkers of ALS.

Sample Type	miRNA Tested	Expression	Influence	Study Group	Author
Blood	miR1915-3p	↓	Inhibition of Bcl-2	6 ALS 10 CTRL	[117]
	miR3665, miR4530, miR4745-5p	↓	NA	18 ALS 16 CTRL	[118]
	miR374B-5p	↓	NA	27 ALS 25 CTRL	[120]
	miR143-3p, miR206 ^&^	↑	^&^ Involved in regeneration of neuromuscular synapses		

ALS—amyotrophic lateral sclerosis patients; CTRL—normal controls; NA—not assessed; ^&^—The influence corresponds only with marked miRNAs.

**Table 4:** Molecular biomarkers of MS.

Sample Type	miRNA Tested	Expression	Influence	Study Group	Author
Blood	miR-125a	↑	B-cell diversification and macrophage activity	40 MS 40 CTRL	[145]
	miR-146b	↑	Dysregulated in autoimmune disorders; widely expressed and involved in the differentiation and activation of various immunocyte lineages: IL-17-producing T cells, Treg cells, monocytes, dendritic cells, macrophages, and B cells		
	miR-200c	↑	Suppresses the expression of the Ets-1 protein—a negative regulator of Th17 differentiation		
	miR-152	↓	Controls the migration and invasive potential of cancer cells		
	miR-145 ^&^, miR-376 c-3p, miR-128-3p, miR-191-5p, miR-26a-5p, miR-320a, miR-486-5p, miR-320b, miR-25-3p, miR-24-3p, miR-140-3p	↑	^&^ Mediates pleiotropic effects of interferon-beta through the mitogen-activated protein kinase signaling pathway		[148,149,150]
	miR-572 ^&^, miR-15b, miR-23a, let-7 c-5p, miR-16, miR-24, miR-137, miR-181	↓	^&^ Targets neural cell adhesion molecule (NCAM)		
	miR-150-5p	↑	Sensor for activation of adaptive immunity associated with the presence of oligoclonal bands	10 MS 10 CTRL	[147]
	miR-155-5p	↑	Promotes differentiation of Th17 and Treg cells, activates Th17 function, activates microglia-mediated immune responses through proinflammatory cytokines, increases permeability of the blood–brain barrier, and enhances leukocyte adhesion to endothelial cells		
	miR-15a-3p, miR-34c-5p	↓	Unknown in MS; proapoptotic activity in cancer		

MS—multiple sclerosis patients; CTRL—normal controls; ^&^—The influence corresponds only with marked miRNAs.

## Data Availability

Not applicable.

## Abbreviations

AD	Alzheimer’s disease
ALS	Amyotrophic lateral sclerosis
Aβ	Amyloid β
BACE1	Beta-site APP-cleaving enzyme 1
BBB	Blood–brain barrier
CCH	Chronic cerebral hypofusion
CIS	Clinically isolated syndrome
CNS	Central nervous system
CSF	Cerebrospinal fluid
DS	Down syndrome
EBV	Epstein–Barr virus
EOAD	Early-onset Alzheimer’s disease
FLC	Immunoglobulin-free light chains
HLA	Human leukocyte antigen
KFLC	Kappa-free light chains
LFLC	Lambda-free light chains
LOAD	Late-onset Alzheimer’s disease
MCI	Mild cognitive impairment
miRNAs	microRNAs
MMPs	Matrix metalloproteinases
MS	Multiple sclerosis
NDs	Neurodegenerative diseases
Nf-L	Neurofilament light chains
NFTs	Neurofibrillary tangles
NF-κB	Nuclear factor κB
NGS	Next-generation deep sequencing
PD	Parkinson’s disease,
pNfH	Phosphorylated neurofilament heavy chains
PPMS	Primary progressive MS
pTau	Phosphorylated tau
pTau181	Tau phosphorylated at threonine 181
ROS	Reactive oxygen species
RRMS	Relapsing–remitting MS
SOD1	Superoxide dismutase 1
SPMS	Secondary progressive MS
TDP-43	Transactive response DNA-binding protein 43 kDa
tTau	Total tau protein
